# *IFNG* +874A/T Polymorphism Among Asymptomatic HTLV-1-Infected Individuals Is Potentially Related to a Worse Prognosis

**DOI:** 10.3389/fmicb.2018.00795

**Published:** 2018-05-18

**Authors:** Maria A. F. Queiroz, Vânia N. Azevedo, Ednelza da S. G. Amoras, Tuane C. F. Moura, Marluísa de O. Guimarães Ishak, Ricardo Ishak, Antonio C. R. Vallinoto, Rosimar N. Martins Feitosa

**Affiliations:** Laboratory of Virology, Institute of Biological Sciences, Federal University of Pará, Belém, Brazil

**Keywords:** HTLV-1, IFN-γ, polymorphism, plasma dosage, clinical symptoms

## Abstract

HTLV-1 infections are persistent and frequently latent; however, productive infections trigger different types of immunological responses that utilize cytokines to control infection. The present study investigated the role of *IFNG* +874A/T polymorphisms among 153 HTLV-1-infected individuals (33 clinically diagnosed with TSP/HAM, 22 with rheumatologic manifestations, 2 with dermatitis, 1 with uveitis, and 95 asymptomatic patients) and 300 healthy control individuals. Genotyping and proviral HTLV-1 load assessment were performed using real-time PCR assays, and the plasma levels of IFN-γ were measured using an enzyme immunoassay (ELISA). Genotype frequencies were not significantly different, but the presence of the T allele was higher (*p* < 0.0142) among the asymptomatic patients. Plasma levels of IFN-γ were significantly higher (*p* < 0.0137) among those with the TT genotype. Their proviral load was also higher, although this elevation did not reach statistical significance. There was no difference in the IFN-γ plasma levels among the symptomatic patients, even when ranked according to disease severity (TSP/HAM or rheumatologic manifestations). However, the difference among asymptomatic patients with the T allele was significantly higher (*p* < 0.0016) and similar to the plasma levels observed among symptomatic individuals. These results suggest that the *IFNG* +874A/T polymorphism may modulate the plasma levels of IFN-γ during HTLV-1 infection. Asymptomatic carriers of the polymorphic genotypes appear to develop an inflammatory response in a shorter timeframe, triggering progression to HTLV-1-related symptoms and disorders. These results further suggest that HTLV-1-infected asymptomatic individuals expressing the *IFNG* +874A/T polymorphism should be monitored more closely in order to readily detect the increase in clinical symptoms, as these patients are potentially at risk of a poor prognosis and should therefore start available treatment procedures earlier.

## Introduction

Human T-cell lymphotropic virus type 1 (HTLV-1) infects approximately 5–10 million people worldwide (Cassar and Gessain, 2017). The most relevant endemic regions are located in southeastern Japan, sub-Saharan Africa, the Caribbean, the Middle East, the Austro-Melanesia region, and South America (Gessain and Cassar, 2012). Brazil is an important endemic area for the virus, and a great diversity of diseases associated with HTLV-1 have been observed there (Pombo de Oliveira et al., 1999; Rathsam-Pinheiro et al., 2009; Grassi et al., 2011; Okajima et al., 2013). The prevalence of infection varies among different regions of the country: the southern region usually shows the lowest rates, and the northeast region shows the highest rates (Catalan-Soares et al., 2005).

Most HTLV-1 infections are asymptomatic; however, under certain conditions not yet fully understood, the virus may lead to the development of associated diseases, including HTLV-1-associated myelopathy/tropical spastic paraparesis (HAM/TSP), adult T-cell leukemia/lymphoma (ATLL), and inflammatory syndromes such as rheumatoid arthritis, dermatitis, and uveitis (Yakova et al., 2005; Okajima et al., 2013; Quaresma et al., 2015). The development of symptoms, particularly those of HAM/TSP and rheumatoid arthritis, are associated with a high proviral load in the peripheral blood and dysregulation of the immunological response against the virus (Grassi et al., 2011; Coutinho et al., 2014; da Silva Dias et al., 2016). Several studies have investigated the mechanisms underlying how immunological factors may change the course of HTLV-1 infection (Goon et al., 2004; Kato et al., 2004; Yakova et al., 2005; Best et al., 2009; Araya et al., 2014; Coutinho et al., 2014). The virus induces spontaneous proliferation of T CD4^+^ and T CD8^+^ lymphocytes and natural killer cells (Goon et al., 2004; Pinto et al., 2011). The increases in the numbers of these cells may lead to the development of a hyperimmune response and the marked production of proinflammatory cytokines, contributing to the pathogenesis of inflammatory disorders associated with HTLV-1 (Montanheiro et al., 2009; Yamano et al., 2009).

Genetic variations in important components of the immunological system are associated with the presence of symptoms in HTLV-1 infection (Vallinoto et al., 2012; Shirdel et al., 2013; de Sá et al., 2016). IFN-γ is the main proinflammatory cytokine associated with clinical symptoms etiologically linked to HTLV-1 infection (Montanheiro et al., 2009; da Silva Dias et al., 2016). The *IFNG* gene expresses CA-repeat microsatellite polymorphisms, and the major one—*IFNG* +874 A/T—is associated with the increased production of IFN-γ (Pravica et al., 1999). The presence of this polymorphism has been associated with multiple viral infections, including HIV-1, hepatitis B virus (Freitas et al., 2015; Al Kadi and Monem, 2017) and susceptibility to HTLV-1 infection (Rocha-Júnior et al., 2012).

The direct influence of this polymorphism on IFN-γ levels in HTLV-1-infected individuals has not reportedly been characterized. The marked inflammatory response observed with HTLV-1-associated diseases was therefore investigated in order to determine the influence of the *IFNG* +874 A/T polymorphism on the plasma level of IFN-γ and its relationship with the progression of HTLV-1 infection to symptomatic disease.

## Materials and Methods

### Study Population

This study examined 153 HTLV-1-infected individuals (33 clinically diagnosed with HAM/TSP, 22 with rheumatic manifestations, 2 with dermatitis, 1 with uveitis, and 95 asymptomatic individuals) of both sexes, older than 18 years, not currently being treated with glucocorticoids, who were followed in the outpatient clinic of the Tropical Medicine Division of the Federal University of Pará. Clinical and laboratory criteria were used to diagnose the diseases associated with HTLV-1 according to the Brazilian Guidelines for HTLV-1 diseases from the Brazilian Ministry of Health (Brasil. Ministério da Saúde, 2013). The control group consisted of 300 blood donors from the Center for Hemotherapy and Hematology of Pará (HEMOPA) who were used to compare the genotype and allele frequencies of the *IFNG* +874 A/T polymorphism. The control group was matched by age and sex with the HTLV-1-infected individuals.

### Collection and Storage of Samples

Blood samples were collected (10 mL) by intravenous puncture using a vacuum collection system containing ethylenediaminetetraacetic acid (EDTA) as an anticoagulant. The samples were centrifuged at 3,000 rpm for 10 min to separate the leukocytes. Leukocytes were used to extract genomic DNA for analysis of the *IFNG* +874 A/T polymorphism and quantification of the proviral load. Plasma samples were used for the quantification of IFN-γ. Samples were stored at -70°C until use.

### Laboratory Tests

#### DNA Extraction

DNA was extracted from peripheral blood leukocytes using a Puregene kit (Puregene, Gentra Systems, Inc., United States) according to the manufacturer’s protocol, which included cell lysis, protein precipitation, DNA precipitation, and hydration. After extraction, the DNA was quantified using a Qubit^®^ 2.0 fluorometer (Life Technologies, Carlsbad, CA, United States) and the Qubit^TM^ DNA Assay Kit (Life Technologies, Carlsbad, CA, United States) reagents, following the manufacturer’s protocol.

#### Quantification of the Proviral Load of HTLV-1

Proviral load was quantified by qPCR using three target sequences synthesized using the TaqMan^®^ system (Life Technologies, Foster City, CA, United States), according to a previously described protocol by Tamegão-Lopes et al. (2006), namely, collection of 5 mL of whole blood for DNA extraction from leukocytes, followed by relative quantification using real-time PCR. The obtained results were further adjusted to an absolute proviral quantification by considering the leukocyte counts per mm^3^, and the results were expressed as DNA proviral copies/mm^3^.

#### Genotyping of *IFNG*+874 A/T (rs2430561)

*IFNG* +874 A/T polymorphism located in the first intron of the gene encoding IFN-γ was analyzed by real-time PCR using a StepOnePLUS^TM^ Real-Time PCR system. The specific primers (IFNG-F: 5′-TTC AGA CAT TCA CAA TTG ATT TTA TTC T-3′ and IFNG-R: 5′-CCC CCA ATG GTA CAG GTT TC-3′) and probes (FAM-AAAATCAAATCTCACACACACA-MGB and VIC-AAAATCAAATCACACACACACA-MGB) were previously described (Tso et al., 2005). The reaction followed a program of 10 min at 95°C, 40 cycles of 15 s at 95°C and 1 min at 60°C.

#### Quantification of Plasma IFN-γ Levels

Plasma IFN-γ levels were measured by the Ready-SET-Go^®^ enzyme-linked immunosorbent assay (ELISA) (eBioscience, San Diego, CA, United States), which uses specific monoclonal antibodies to detect the cytokine following the manufacturer’s instructions.

### Statistical Analysis

Genotype and allele frequencies were estimated by direct counting. Significant differences between groups were determined using the chi-squared test. Hardy–Weinberg equilibrium was calculated to evaluate whether the distribution of the genotype frequencies observed was in agreement with the expected frequencies. Plasma IFN-γ levels were compared between groups using the non-parametric Mann–Whitney test. All tests were performed using the software BioEstat 5.3 (Ayres et al., 2011). Statistical associations at *p-values* < 0.05 were considered statistically significant.

### Ethical Considerations

The project was approved by the Research Ethics Committee of the João de Barros Barreto University Hospital of the Federal University of Pará (protocol no. 2061/2005). All study participants were fully informed of the research objectives, and those who agreed to participate signed an informed consent form.

## Results

The majority of HTLV-1-infected individuals were female (69.9%, 107/153), with a mean age of 50.3 years. The frequency of the wild-type genotype was higher in the infected group, but there was no significant difference between the genotype and allele frequencies when compared to the control group (**Table 1**).

**Table 1:** Genotype and allele frequencies of the *IFNG* +874 A/T polymorphism among HTLV-1-infected individuals and the control group.

Genotypes and alleles	HTLV	Control	*p^∗^*
	*n* (%)	*n* (%)	
AA	83 (54.25)	174 (58.00)	0.5153
AT	58 (37.91)	110 (36.67)	
TT	12 (7.84)	16 (5.33)	
^∗^A	0.73	0.76	0.7456
^∗^T	0.27	0.24	


*n, number of individuals; ^∗^chi-squared (χ^*2*^) test.*

Comparison of genotype frequencies between infected (asymptomatic) and diseased (symptomatic) individuals showed no significant differences. However, the presence of the T allele was significantly higher (*p* = 0.0142) among asymptomatic individuals than among diseased patients (**Table 2**).

**Table 2:** Genotype and allele frequencies of the *IFNG* +874 A/T polymorphism among symptomatic and asymptomatic HTLV-1-infected individuals.

Genotypes and alleles	Symptomatic	Asymptomatic	*p^∗^*
	*n* (%)	*n* (%)	
AA	34 (58.63)	49 (51.58)	0.6689
AT	19 (32.75)	39 (41.05)	
TT	5 (8.62)	7 (7.37)	
^∗^A	0.87	0.72	0.0142
^∗^T	0.13	0.28	


*n, number of individuals; ^∗^chi-squared (χ^*2*^) test.*

**FIGURE 1:**
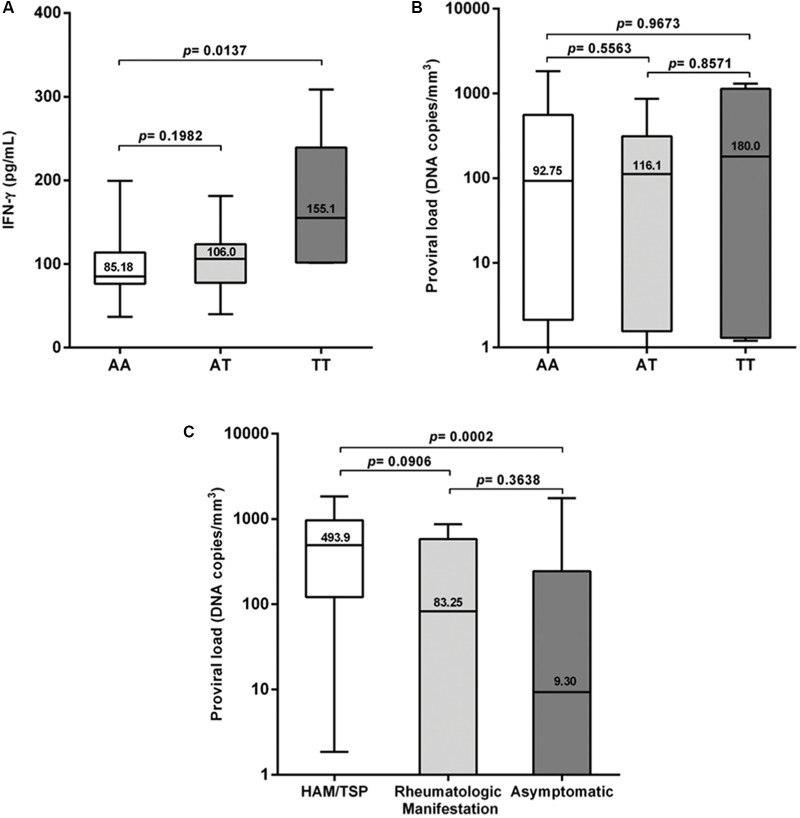
**(A)** IFN-γ plasma levels classified according to *IFNG* +874 A/T genotypes, **(B)** proviral load among HTLV-1-infected individuals expressing *IFNG* +874 A/T genotypes, and **(C)** proviral load classified according to the presence or absence of symptoms. Mann–Whitney test.

**FIGURE 2:**
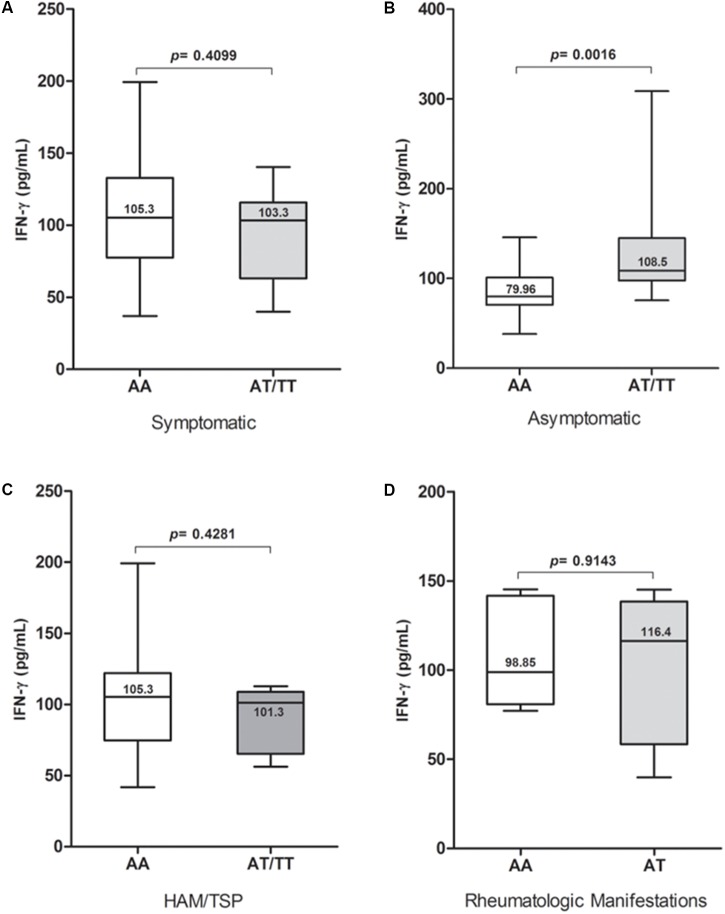
IFN-γ plasma levels among **(A)** symptomatic and **(B)** asymptomatic HTLV-1-infected individuals carrying *IFNG* +874 A/T genotypes, as well as **(C,D)** according to the presence and presentation of disease. Mann–Whitney test.

Plasma IFN-γ levels were significantly higher (*p* = 0.0137) in the group with the TT genotype (**Figure 1A**); they also showed a higher proviral load, although this difference was not statistically significant when compared with the other genotypes (**Figure 1B**). The proviral load was significantly higher (*p* = 0.0002) among the HAM/TSP patients than among asymptomatic individuals (**Figure 1C**). No significant difference in plasma levels was observed between patients with the wild-type genotype and those with the polymorphic allele in the symptomatic group (**Figure 2A**), and no significant difference was observed when comparing patients with the wild-type genotype and those with TSP/HAM or those with rheumatic disease (**Figures 2C,D**). However, there was a significant difference among the asymptomatic HTLV-1-infected individuals: IFN-γ plasma levels among patients with the T allele were significantly higher (*p* = 0.0016) and reached values that were similar to those exhibited by symptomatic individuals (**Figure 2B**).

## Discussion

The immunological response elicited by HTLV-1-infected individuals appears to be influenced and modulated by the virus, as its presence may promote the spontaneous proliferation of T CD4^+^ and T CD8^+^ lymphocytes and induce the production of proinflammatory cytokines responsible for the symptoms of various diseases, such as HAM/TSP (Goon et al., 2002). Along with other cytokines involved in this process, IFN-γ is likely the most important in the immunological pathogenesis of HAM/TSP (Montanheiro et al., 2009), and genetic alterations in its gene sequence lead to an exacerbated inflammatory process and an increase in the severity of the disease.

In the present study, the *IFNG* +874 A/T polymorphism was not associated with susceptibility to HTLV-1 infection; there was no significant difference in genotype or allele frequencies between HTLV-1-infected individuals and those in the control group. However, the polymorphic allele was associated with the absence of symptoms in HTLV-1-infected individuals. The wild-type allele was already associated as a risk factor for HIV-1 infection (Freitas et al., 2015) and for the disease progression of hepatitis B (Al Kadi and Monem, 2017). The results obtained herein with the allele frequencies suggest that the T allele would act as a protective factor against the progression to disease among HTLV-1-infected individuals.

These results were not correlated with IFN-γ levels measured in plasma. Plasma IFN-γ levels were significantly higher among individuals carrying the *IFNG* +874 T allele (genotypes AT and TT); these individuals also showed a higher proviral load, although the differences were not statistically significant. The presence of the polymorphism may increase the levels of inflammation and lead to disease progression. Elevated IFN-γ levels in patients with HAM/TSP have previously been associated with central nervous system inflammatory disorders (Yamano et al., 2009).

Proviral load was higher in the patient group; however, in contrast to a previous report from Yakova et al. (2005), the plasma proviral loads of patients with rheumatic disease were higher than those of asymptomatic individuals but lower than those of TSP/HAM patients. Statistical significance was achieved only when HAM/TSP patients were compared with asymptomatic carriers of the virus.

The polymorphism did not affect plasma IFN-γ levels among symptomatic patients when they were assessed according to the clinical presentation of the disease or in the presence of the enhanced inflammatory process involving the nervous tissue and joints, as observed among patients with HAM/TSP and rheumatoid arthritis. Several other factors have been associated with the severity of disease, which may be more relevant than the presence of the *IFNG* +874 A/T polymorphism in this situation (Ahuja et al., 2007; Quaresma et al., 2015).

A significantly different situation was observed with the comparison of IFN-γ levels in the asymptomatic group. Individuals carrying the polymorphic allele showed significantly higher levels of IFN-γ than those carrying the wild-type allele. This may represent a previously unidentified risk factor for disease progression, as these infected individuals show IFN-γ levels similar to those of symptomatic individuals. The proviral load was occasionally detected, and the increase in IFN-γ levels may serve as a safe, reliable immunological marker of a poor prognosis and the evolution of disease pathogenesis, which may indicate clinical progression to HAM/TSP (Montanheiro et al., 2009; da Silva Dias et al., 2016).

Although the proviral load is reportedly elevated among HTLV-1-infected individuals with neurological dysfunction (Silva et al., 2007; Grassi et al., 2011), several asymptomatic individuals may present a pattern of immunological response that is associated with a high proviral load (Coutinho et al., 2014) and an inflammatory response similar to those with HAM/TSP (Santos et al., 2004).

Another investigation of the *IFNG* +874 A/T polymorphism, albeit one that did not measure the intensity of the inflammatory process (using the IFN-γ levels as a marker of inflammation), showed that the AT genotype was associated with a higher proviral load (Rocha-Júnior et al., 2012). This suggests that asymptomatic HTLV-1-infected individuals with the T allele of *IFNG* +874 A/T and a high proviral load have a high probability of developing HTLV-1-associated inflammatory diseases.

The immunological response during HTLV-1 infection is complex, and although IFN-γ is crucial in fighting intracellular viral agents by blocking their replication (Schoenborn and Wilson, 2007), elevated levels of this cytokine are harmful to the host; in the case of HTLV-1, this may lead to the progression of severe diseases, including HAM/TSP.

## Conclusion

The present results suggest that the *IFNG* +874 A/T polymorphism may influence IFN-γ plasma levels upon HTLV-1 infection. Asymptomatic individuals carrying the T allele appear to be more likely to develop inflammation more rapidly, which could lead to the onset of associated diseases. The present investigation identifies IFN-γ levels and its sequence polymorphism as an important biomarker to be further investigated and monitored among HTLV-1-infected patients as part of the routine follow-up of asymptomatic individuals, as they are potentially at risk of developing a worse prognosis of disease and should start available treatment procedures earlier.

## Author Contributions

MQ, RI, AV, and RM designed the study, analyzed and interpreted the data. MQ, VA, EA, and TM performed the experiments. MQ wrote the manuscript. MG, RI, and AV oversaw the experiments and edited the manuscript. MQ, VA, EA, TM, MG, RI, AV and RM reviewed the manuscript.

## Conflict of Interest Statement

The authors declare that the research was conducted in the absence of any commercial or financial relationships that could be construed as a potential conflict of interest.
